# Complete genome sequence of *Vulcanisaeta distributa* type strain (IC-017^T^)

**DOI:** 10.4056/sigs.1113067

**Published:** 2010-09-28

**Authors:** Konstantinos Mavromatis, Johannes Sikorski, Elke Pabst, Hazuki Teshima, Alla Lapidus, Susan Lucas, Matt Nolan, Tijana Glavina Del Rio, Jan-Fang Cheng, David Bruce, Lynne Goodwin, Sam Pitluck, Konstantinos Liolios, Natalia Ivanova, Natalia Mikhailova, Amrita Pati, Amy Chen, Krishna Palaniappan, Miriam Land, Loren Hauser, Yun-Juan Chang, Cynthia D. Jeffries, Manfred Rohde, Stefan Spring, Markus Göker, Reinhard Wirth, Tanja Woyke, James Bristow, Jonathan A. Eisen, Victor Markowitz, Philip Hugenholtz, Hans-Peter Klenk, Nikos C. Kyrpides

**Affiliations:** 1 DOE Joint Genome Institute, Walnut Creek, California, USA; 2 DSMZ - German Collection of Microorganisms and Cell Cultures GmbH, Braunschweig, Germany; 3 University of Regensburg, Microbiology – Archaeenzentrum. Regensburg, Germany; 4 Los Alamos National Laboratory, Bioscience Division, Los Alamos, New Mexico, USA; 5 Biological Data Management and Technology Center, Lawrence Berkeley National Laboratory, Berkeley, California, USA; 6 Oak Ridge National Laboratory, Oak Ridge, Tennessee, USA; 7 HZI – Helmholtz Centre for Infection Research, Braunschweig, Germany; 8 University of California Davis Genome Center, Davis, California, USA

**Keywords:** hyperthermophilic, acidophilic, non-motile, microaerotolerant anaerobe, *Thermoproteaceae*, *Crenarchaeota*, GEBA

## Abstract

*Vulcanisaeta distributa* Itoh *et al.* 2002 belongs to the family *Thermoproteaceae* in the phylum *Crenarchaeota*. The genus *Vulcanisaeta* is characterized by a global distribution in hot and acidic springs. This is the first genome sequence from a member of the genus *Vulcanisaeta* and seventh genome sequence in the family *Thermoproteaceae*. The 2,374,137 bp long genome with its 2,544 protein-coding and 49 RNA genes is a part of the *** G****enomic* *** E****ncyclopedia of* *** B****acteria* *and* *** A****rchaea * project.

## Introduction

Strain IC-017^T^ (= DSM 14429 = JCM 11212) is the type strain of the species *Vulcanisaeta distributa*, which is the type species of its genus *Vulcanisaeta* [1]. The only other species in the genus is *V. souniana* [1,2]. The genus name derives from the Latin words ‘*vulcanicus*’ meaning volcanic, and ‘*saeta*’ meaning stiff hair, to indicate a rigid rod inhabiting volcanic hot springs [1]. The species epithet derives from the Latin ‘*distributa*’, referring to the wide distribution of strains belonging to this species [1]. The type strain IC-017^T^ was isolated from a hot spring in Ohwakudani, Kanagawa, Japan [1]. Fourteen additional strains [IC-019, IC-029 (= JCM 11213), IC-030, IC-032, IC-051, IC-052, IC-058, IC-064 (= JCM 11214), IC-065 (= JCM 11215), IC-124 (= JCM 11216), IC-135 (= JCM 11217), IC-136, IC-140 and IC-141 (= JCM 11218)] are included in this species [1]. At the time of the species description, the terminus ‘distributa’ referred simply to a wide distribution within Japan [1]. However, 16S rRNA sequences which probably belong to the genus *Vulcanisaeta* (≥95% sequence similarity to *V. distributa*) have been obtained from 117°C hot deep-sea hydrothermal fluid in the south Mariana area [3]. Clone sequences that are highly similar to the 16S rRNA gene sequence of strain IC-017^T^ were obtained from an acidic hot spring water at the Tatung Volcano area in Northern Taiwan (99%, FJ797325), the hot Sylvan Spring in Yellowstone National Park (=YNP, USA, 98%, DQ243774), at the Cistern Hot Spring at Norris Geyser Basin in YNP (98%, DQ924709) and also at other springs in YNP (98%, DQ833773). Metagenomic sequences from uncultured clones in YNP (94%, ADKH01000984) also support these observations. The 16S rRNA gene similarity values to non-hot-spring metagenomes, e.g., from marine, soil, or human gut, were all below 83%, indicating that *Vulcanisaeta* is probably not found in these habitats (status July 2010).

Although it is not the case for the type strain IC-017^T^, *V. distributa* recently received further interest, as it was found that strain IC-065 contained a 691 bp large intron within its 16S rRNA sequence [4]. Novel 16S rRNA introns have been found in several members of the family *Thermoproteaceae* [4]. Here we present a summary classification and a set of features for *V. distributa* strain IC-017^T^, together with the description of the complete genomic sequencing and annotation.

## Classification and features

**Figure 1:**
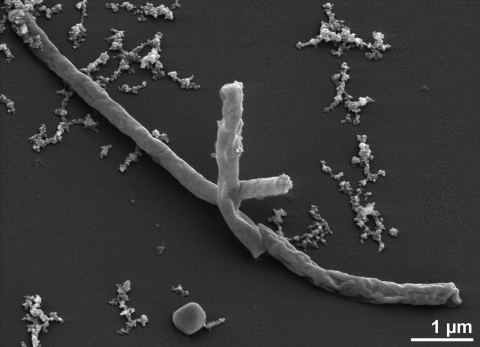
Scanning electron micrograph of *V. distributa* IC-017^T^

The cells of strain IC-017^T^ are rigid, straight to slightly curved rods (Figure 1 and Table 1)[4]. Occasionally, they bend, branch out, or bear spherical bodies at the terminae (not seen in Figure 1), which have been termed as 'golf clubs'. Most cells are 0.4-0.6 µm in width and 3-7 µm long [4]. Pili have been observed to rise terminally or laterally; motility has not been observed [4]. Usually, strain IC-017^T^ grows anaerobically. However, when cultured in media in which sulfur is replaced by sodium thiosulfate (1.0 g/l), strain IC-017^T^ showed weak growth in a low-oxygen atmosphere (1%), but not in air [4].

**Table 1:** Classification and general features of V. distributa IC-017T according to the MIGS recommendations [5]

**MIGS ID**	**Property**	**Term**	**Evidence code**
	Current classification	Domain *Archaea*	TAS [6]
		Phylum *Crenarchaeota*	TAS [7,8]
		Class *Thermoprotei*	TAS [8,9]
		Order *Thermoproteales*	TAS [10-13]
		Family *Thermoproteaceae*	TAS [10,12,13]
		Genus *Vulcanisaeta*	TAS [1]
		Species *Vulcanisaeta distributa*	TAS [1]
		Type strain IC-017	TAS [1]
	Gram stain	not reported	TAS [1]
	Cell shape	rigid, straight to slightly curved rods	TAS [1]
	Motility	non-motile	TAS [1]
	Sporulation	not reported	TAS [1]
	Temperature range	70-92°C	TAS [1]
	Optimum temperature	90°C	TAS [1]
	Salinity	1% NaCl or below	TAS [1]
MIGS-22	Oxygen requirement	microaerotolerant anaerobe	TAS [1]
	Carbon source	yeast extract, peptone, beef extract, casamino acids, gelatin, maltose, starch, malate, galactose, mannose	TAS [1]
	Energy source	heterotrophic	TAS [1]
MIGS-6	Habitat	acidic hot environments (water, soil, mud)	TAS [1]
MIGS-15	Biotic relationship	free living	TAS [1]
MIGS-14	Pathogenicity	not pathogenic	NAS
	Biosafety level	1	TAS [14]
	Isolation	acidic hot water	TAS [1]
MIGS-4	Geographic location	Ohwakudani, Japan	TAS [1]
MIGS-5	Sample collection time	September 1993	TAS [1]
MIGS-4.1 MIGS-4.2	Latitude Longitude	35.447 139.642	NAS
MIGS-4.3	Depth	unknown	
MIGS-4.4	Altitude	unknown	

Evidence codes - IDA: Inferred from Direct Assay (first time in publication); TAS: Traceable Author Statement (i.e., a direct report exists in the literature); NAS: Non-traceable Author Statement (i.e., not directly observed for the living, isolated sample, but based on a generally accepted property for the species, or anecdotal evidence). These evidence codes are from of the Gene Ontology project [15]. If the evidence code is IDA, then the property was directly observed by one of the authors or an expert mentioned in the acknowledgements.

In contrast to *Thermocladium* or *Caldivirga* strains, *V. distributa* grows well even in the absence of a vitamin mixture or archaeal cell-extract solution in the medium [4]. All seven tested strains of *V. distributa* were shown to be resistant to chloramphenicol, kanamycin, oleandomycin, streptomycin and vancomycin, but sensitive to erythromycin, novobiocin and rifampicin (all at 100 µg per ml) [4]. *V. distributa* needs acidic conditions to grow (pH 3.5 to 5.6). Under optimal growth conditions, the doubling time is 5.5 to 6.5 hours [4]. Sulfur or thiosulfate is required as an electron acceptor. Strain IC-017^T^ does not utilize D-arabinose, D-fructose, lactose, sucrose, D-xylose, acetate, butyrate, formate, fumarate, propionate, pyruvate, succinate, methanol, formamide, methylamine or trimethylamine as carbon sources and does not utilize fumarate, malate or nitrate as electron acceptors [4].

**Figure 2:**
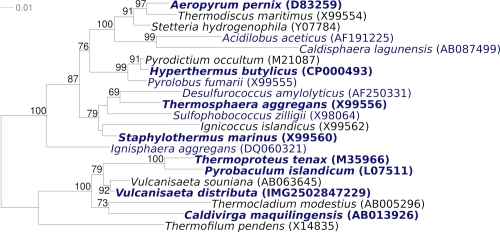
Phylogenetic tree highlighting the position of *V. distributa* IC-017^T^ relative to the other type strains within the genus *Vulcanisaeta* and the type strains of the other genera within *Thermoproteales*. The tree was inferred from 1,356 aligned characters [16,17] of the 16S rRNA gene sequence under the maximum likelihood criterion [18] and rooted with the type strains of the genera of *Desulfurococcales* and *Acidilobales*. The branches are scaled in terms of the expected number of substitutions per site. Numbers above branches are support values from 150 bootstrap replicates [19] if larger than 60%. Lineages with type strain genome sequencing projects registered in GOLD [20] are shown in blue, published genomes [21-24] and INSDC accessions CP000504 and CP00852 in bold.

Figure 2 shows the phylogenetic neighborhood of *V. distributa* IC-017^T^ in a 16S rRNA based tree. The sequence of the single 16S rRNA gene copy in the genome of strain IC-017^T^ does not differ from the previously published 16S rRNA sequence (AB063630).

### Chemotaxonomy

Strain IC-017^T^ possesses cyclic and acyclic tetraether core lipids [4]. The major cellular polyamines are norspermidine (1.25), spermidine (0.55), agmatine (0.15), norspermine (0.1) and cadaverine (0.1) (values are in µmol/g wet weight of the cell) [25]. Further chemotaxonomic data are not available.

## Genome sequencing and annotation

### Genome project history

This organism was selected for sequencing on the basis of its phylogenetic position [26], and is part of the *** G****enomic* *** E****ncyclopedia of* ** B**acteria *and* *** A****rchaea * project [27]. The genome project is deposited in the Genome OnLine Database [20] and the complete genome sequence is deposited in GenBank. Sequencing, finishing and annotation were performed by the DOE Joint Genome Institute (JGI). A summary of the project information is shown in Table 2.

**Table 2:** Genome sequencing project information

**MIGS ID**	**Property**	**Term**
MIGS-31	Finishing quality	Finished
MIGS-28	Libraries used	Two genomic libraries: one 454 pyrosequence standard library, one 454 PE library (22.9kb insert size)
MIGS-29	Sequencing platforms	454 GS FLX Titanium
MIGS-31.2	Sequencing coverage	106.3 × pyrosequence
MIGS-30	Assemblers	Newbler version 2.0.0-PostRelease- 09/05/2008, phrap
MIGS-32	Gene calling method	Prodigal 1.4, GenePRIMP
	INSDC ID	CP002100
	Genbank Date of Release	September 23, 2010
	GOLD ID	Gc01374
	NCBI project ID	32589
	Database: IMG-GEBA	2502790013
MIGS-13	Source material identifier	DSM 14429
	Project relevance	Tree of Life, GEBA

### Growth conditions and DNA isolation

*V. distributa* IC-017^T^, DSM 14429, was grown anaerobically in DSMZ medium 88 (*Sulfolobus* medium) [28] at 90°C. DNA was isolated from 0.5-1 g of cell paste using Qiagen Genomic 500 DNA Kit (Qiagen, Hilden, Germany) following the standard protocol as recommended by the manufacturer.

### Genome sequencing and assembly

The genome was sequenced using a 454 sequencing platform. All general aspects of library construction and sequencing can be found at the JGI website (http://www.jgi.doe.gov/). Pyrosequencing reads were assembled using the Newbler assembler version 2.0.0-PostRelease-09/05/2008 (Roche). The initial Newbler assembly consisted of 147 contigs in 13 scaffolds and was converted into a phrap assembly by making fake reads from the consensus, and collecting the read pairs in the 454 paired end library. Draft assemblies were based on 252.4 Mb 454 draft and all of the 454 paired end data. Newbler parameters are -consed -a 50 -l 350 -g -m -ml 20.

The Phred/Phrap/Consed software package (www.phrap.com) was used for sequence assembly and quality assessment in the following finishing process. After the shotgun stage, reads were assembled with parallel phrap (High Performance Software, LLC). Possible mis-assemblies were corrected with gapResolution (http://www.jgi.doe.gov/), Dupfinisher [29], or sequencing cloned bridging PCR fragments with subcloning or transposon bombing (Epicentre Biotechnologies, Madison, WI) [30]. Gaps between contigs were closed by editing in Consed, by PCR and by Bubble PCR primer walks (J.-F.Chang, unpublished). A total of 97 additional reactions were necessary to close gaps and to raise the quality of the finished sequence. The error rate of the completed genome sequence is less than 1 in 100,000. The final assembly contains  0.8 million pyrosequencing reads that provide 106.3 x coverage of the genome.

### Genome annotation

Genes were identified using Prodigal [31] as part of the Oak Ridge National Laboratory genome annotation pipeline, followed by a round of manual curation using the JGI GenePRIMP pipeline [32]. The predicted CDSs were translated and used to search the National Center for Biotechnology Information (NCBI) nonredundant database, UniProt, TIGRFam, Pfam, PRIAM, KEGG, COG, and InterPro databases. Additional gene prediction analysis and functional annotation was performed within the Integrated Microbial Genomes - Expert Review (IMG-ER) platform [33].

## Genome properties

**Figure 3:**
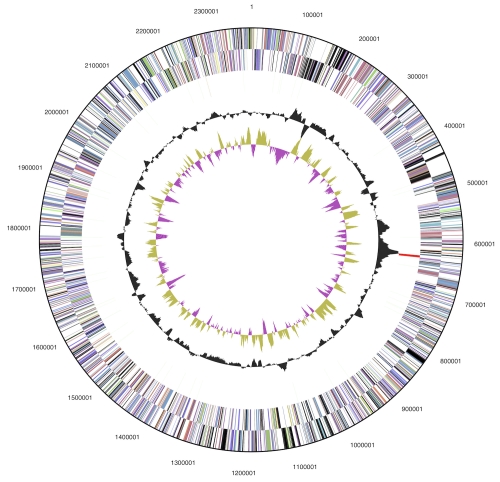
Graphical circular map of the genome. From outside to the center: Genes on forward strand (color by COG categories), Genes on reverse strand (color by COG categories), RNA genes (tRNAs green, rRNAs red, other RNAs black), GC content, GC skew.

The genome consists of a 2,374,137 bp long chromosome with a 45.4% GC content (Table 3 and Figure 3). Of the 2,593 genes predicted, 2,544 were protein-coding genes, and 49 RNAs; fifty one pseudogenes were also identified. The majority of the protein-coding genes (57.2%) were assigned a putative function while the remaining ones were annotated as hypothetical proteins. The distribution of genes into COGs functional categories is presented in Table 4.

**Table 3:** Genome Statistics

**Attribute**	**Value**	**% of Total**
Genome size (bp)	2,374,137	100.00%
DNA coding region (bp)	2,136,210	98.11%
DNA G+C content (bp)	1,078,516	45.43%
Number of replicons	1	
Extrachromosomal elements	0	
Total genes	2,593	100.00%
RNA genes	49	1.89%
rRNA operons	1	
Protein-coding genes	2,544	98.11%
Pseudo genes	51	1.97%
Genes with function prediction	1,483	57.19%
Genes in paralog clusters	327	12.61%
Genes assigned to COGs	1,548	59.70%
Genes assigned Pfam domains	1,665	64.21%
Genes with signal peptides	205	7.91%
Genes with transmembrane helices	591	22.79%
CRISPR repeats	18	

**Table 4:** Number of genes associated with the general COG functional categories

**Code**	**Value**	**%age**	**Description**
J	161	9.6	Translation, ribosomal structure and biogenesis
A	4	0.2	RNA processing and modification
K	65	3.9	Transcription
L	67	4.0	Replication, recombination and repair
B	4	0.2	Chromatin structure and dynamics
D	18	1.1	Cell cycle control, cell division, chromosome partitioning
Y	0	0.0	Nuclear structure
V	13	0.8	Defense mechanisms
T	25	1.5	Signal transduction mechanisms
M	61	3.6	Cell wall/membrane/envelope biogenesis
N	8	0.5	Cell motility
Z	1	0.1	Cytoskeleton
W	0	0.0	Extracellular structures
U	20	1.2	Intracellular trafficking and secretion, and vesicular transport
O	64	3.8	Posttranslational modification, protein turnover, chaperones
C	167	10.0	Energy production and conversion
G	96	5.7	Carbohydrate transport and metabolism
E	160	9.5	Amino acid transport and metabolism
F	55	3.3	Nucleotide transport and metabolism
H	64	3.8	Coenzyme transport and metabolism
I	69	4.1	Lipid transport and metabolism
P	59	3.5	Inorganic ion transport and metabolism
Q	29	1.7	Secondary metabolites biosynthesis, transport and catabolism
R	263	15.7	General function prediction only
S	160	9.5	Function unknown
-	1,045	40.3	Not in COGs
